# Research on Cyanobacterial-Bloom Detection Based on Multispectral Imaging and Deep-Learning Method

**DOI:** 10.3390/s22124571

**Published:** 2022-06-17

**Authors:** Ze Song, Wenxin Xu, Huilin Dong, Xiaowei Wang, Yuqi Cao, Pingjie Huang, Dibo Hou, Zhengfang Wu, Zhongyi Wang

**Affiliations:** 1 State Key Laboratory of Industrial Control Technology, College of Control Science and Engineering, Zhejiang University, Hangzhou 310027, China; sxzturbo@zju.edu.cn (Z.S.); xuwen_xin@zju.edu.cn (W.X.); 22032103@zju.edu.cn (H.D.); xw_wang@zju.edu.cn (X.W.); yuqicao1994@gmail.com (Y.C.); huangpingjie@zju.edu.cn (P.H.); 2 City Intelligence, Cloud & AI, Huawei Technologies Co., Ltd., Shenzhen 518100, China; wuzhengfang@huawei.com (Z.W.); bigbaby.wang@huawei.com (Z.W.)

**Keywords:** remote-sensing technology, cyanobacterial blooms, vegetation index, deep learning

## Abstract

Frequent outbreaks of cyanobacterial blooms have become one of the most challenging water ecosystem issues and a critical concern in environmental protection. To overcome the poor stability of traditional detection algorithms, this paper proposes a method for detecting cyanobacterial blooms based on a deep-learning algorithm. An improved vegetation-index method based on a multispectral image taken by an Unmanned Aerial Vehicle (UAV) was adopted to extract inconspicuous spectral features of cyanobacterial blooms. To enhance the recognition accuracy of cyanobacterial blooms in complex scenes with noise such as reflections and shadows, an improved transformer model based on a feature-enhancement module and pixel-correction fusion was employed. The algorithm proposed in this paper was implemented in several rivers in China, achieving a detection accuracy of cyanobacterial blooms of more than 85%. The estimate of the proportion of the algae bloom contamination area and the severity of pollution were basically accurate. This paper can lay a foundation for ecological and environmental departments for the effective prevention and control of cyanobacterial blooms.

## 1. Introduction

Water is the source of life and an important resource for the survival and development of human beings. However, with increasing human activities and climatic changes, water resources have experienced unprecedented threats, including nutrient enrichment, and inorganic and organic pollution [1]. Water eutrophication has been occurring frequently with the intensification of nitrogen and phosphorus pollution, and one of the severely disastrous consequences is the globally increasing frequency of cyanobacterial blooms [2]. In recent years, a large number of cyanobacterial blooms have erupted in worldwide rivers and lakes, such as Taihu Lake and Dianchi Lake in China, Lake Erie in the USA, and Wood Lake in Canada [3,4,5,6], causing a series of serious ecological problems and posing a huge impact on people’s production and lives. Specifically, cyanobacterial blooms can produce a variety of toxins which cause a range of diseases when ingested by organisms [7]. Moreover, the microbial degradation of cyanobacterial blooms reduce oxygen levels in lakes, resulting in the deaths of fish and inhibiting the growth of aquatic vegetation [8,9]. Mounting evidence shows that cyanobacterial blooms are highly likely to expand further, owing to ongoing eutrophication, in the future [10]. Obviously, it is especially important to perform real-time monitoring and quantification of cyanobacterial blooms.

Currently, routine methods for analyzing cyanobacterial blooms have been widely applied through field sampling and laboratory analysis [11]. However, this traditional method is highly time-consuming, laborious and expensive. Moreover, it cannot obtain comprehensive information on cyanobacterial blooms, so it is not intended for real-time monitoring of large-scale cyanobacterial blooms. Instead, most of the worldwide research results on cyanobacterial-bloom detection are achieved using remote-sensing hyperspectral technology [12]. In these studies, researchers mainly use a variety of vegetation indices to extract spectral features for detecting cyanobacterial blooms, including the Normalized Difference Vegetation Index (NDVI), Atmospherically Resistant Vegetation Index (ARVI), and Enhanced Vegetation Index (EVI) [13,14,15]. In recent years, scholars have also proposed some improved vegetation-index methods to detect cyanobacterial blooms. Hu et al. [16] proposed a new Floating Algae Index (FAI) based on NDVI and EVI to detect floating algae in the open-ocean environment. This approach addresses the sensitivity of traditional vegetation indices to changes in environmental and observational conditions. Huang et al. [17] investigated the variation in chlorophyll concentration distribution in Lake Taihu using the band ratio method, based on Geostationary Ocean Color Imager’s data. Cannizzaro et al. [18] proposed a new quantitative method for cyanobacterial-bloom detection by improving the index based on the analysis of the optical properties of cyanobacterial blooms in Florida Bay. Sachidananda et al. [19] proposed a cyanobacterial index algorithm named CIcyano to detect cyanobacterial blooms in the lakes of eleven states in the United States, and verified the effectiveness of the algorithm.

However, the above applications of vegetation index are all performed with the help of satellite image data, which require a long acquisition period and are inadequate in monitoring low-concentration algal bloom areas in real time. Small UAV remote-sensing technology, which has the advantages of low cost, low risk, high timeliness, and high resolution, is widely used in various scenarios and has become a research hotspot in recent years [20,21,22]. It can effectively make up for the shortcomings of traditional methods, such as long acquisition time period and low detection efficiency. Nevertheless, the vegetation indices mentioned in previous literature are less capable of detecting cyanobacterial blooms in complex river-surface environments, because low-altitude remote-sensing technology will certainly amplify space interference. Moreover, there are still no commercially available preprocessing methods designed for aquatic purposes and UAV close-range remote sensing [20]. Therefore, this paper proposes a new vegetation-index method based on improved feature-band and detail fusion to robustly extract the multispectral features of cyanobacterial blooms using low-altitude airborne detection technology.

In addition, due to the influence of reflections, shadows, and floating objects on the river surface, the detection thresholds of cyanobacterial blooms in different channels are not consistent. Obviously, only using a vegetation index to quantify cyanobacterial blooms is not effective enough, and a more robust algorithm is needed to assist in the detection of cyanobacterial blooms. Deep learning is a powerful and adaptive learning method that can classify or predict non-linear data accurately under complex conditions, which makes it a promising tool in performing image-level semantic segmentation of cyanobacterial blooms. Here, semantic segmentation is necessary and advantageous because of the irregular shape of cyanobacterial blooms. Jonathan et al. [23] from the University of California, Berkeley proposed the Fully Convolutional Network (FCN), pioneering the use of deep learning for image semantic segmentation, but the proposed networks were not sensitive enough to detect details and failed to consider pixels without any spatial coherence. A team from the University of Cambridge developed a deep network for image semantic segmentation named SegNet to segment the regions in an image where objects were located, with accuracy down to the pixel level [24]. Thus far, image semantic segmentation methods based on deep learning have been widely used in autonomous driving, medical image classification and other fields [25,26,27,28], as well as in the detection of cyanobacterial blooms. Yang et al. [29] proposed a deep generative adversarial network (DGAN), which demonstrated a better segmentation result on irregular cyanobacterial-bloom regions. Using an illumination processing algorithm based on a deep neural network (DCNN), Luo et al. [30] normalized the illumination intensity of images to a reasonable range, and thus, effectively improved the accuracy of cyanobacteria detection under the condition of strong light. Xiang et al. [31] adopted the attention module and the number of residual blocks to streamline the structure based on a residual attention network model, and their detection model could discriminate various species of cyanobacterial blooms in Taihu Lake accurately and swiftly.

It is worth noting that few previous studies have paid attention to the phenomenon in which the boundary classifications of cyanobacterial blooms and other objects often give incorrect results. Among the many deep-learning algorithms, the transformer model has a strong model generalization ability. It has a flexible structure and makes essentially no assumptions about the structural errors in the input data, and can be pre-trained to handle large amounts of unlabeled data. More importantly, the attention mechanism will make the semantic association between adjacent pixels better [32]. Therefore, a new transformer model based on feature enhancement and area correction is proposed to overcome the influence of complex environmental factors and boundary misclassification in cyanobacterial-bloom detection.

In this paper, a multispectral detection model for cyanobacterial blooms based on a transformer network is proposed. The spectral features of the multispectral data of cyanobacterial blooms captured by UAV were extracted using an improved vegetation-index method. To classify the feature boundary, feature-enhancement and region-correction modules were added to the original transformer model. The proposed method not only improved the accuracy of detection of cyanobacterial blooms, but also allowed for assessment of the extent of cyanobacterial-bloom pollution and its proportion out of the total size. The results of this study can provide a reference for the control of cyanobacterial blooms in the fields of ecology and the environment.

## 2. Materials and Methods

### 2.1. Data and Evaluation

Data were collected using Phantom 4 multispectral version of the UAV made by DJI. The UAV carries a high-resolution multispectral lens and has six camera sensors, including one color sensor for visible imaging and five monochrome sensors for multispectral imaging in the blue band (B1), green band (B2), red band (B3), red edge-band (B4) and near-infrared band (B5). Healthy and flourishing plants were more reflective in these bands; thus, they can be used to detect cyanobacterial blooms.

The experimental data in this paper were collected from August to October 2021 in several areas of China with severe cyanobacterial blooms, covering many rivers and lakes in Kunming, Yunnan Province; Suzhou, Jiangsu Province; and Huzhou, Zhejiang Province. The data also covered a wide range of weather conditions and external-disturbance scenarios. The dataset included 5400 images and 10 videos, as shown in Table 1.

### 2.2. Methodology

**Figure 1:**
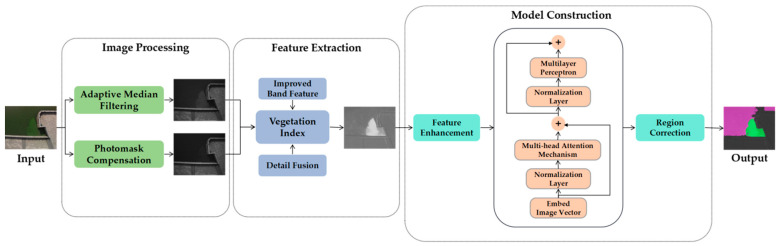
Framework of cyanobacterial-bloom detection algorithm.

In this paper, a cyanobacterial-bloom detection algorithm based on deep learning is proposed. The major steps involved image pre-processing, multi-spectral feature extraction and detection model construction, as shown in Figure 1. Among them, adaptive median filtering and light masking were used in the image pre-processing to remove the background noise generated by the sensor itself and the interference of strong light; an improved vegetation index was used in the feature extraction to extract the spectral features of cyanobacterial blooms more accurately; and a feature-enhancement module and area-correction module were added to address the misclassification of cyanobacterial-bloom boundaries during model construction.

#### 2.2.1. Image Processing

Due to the influence of various environmental factors, multi-dimensional random noise, which interfered with the feature extraction of cyanobacterial blooms, existed in the obtained multi-spectral image data. Aimed at noises of different sizes and shapes in the images, adaptive median filtering was used to improve signal-to-noise ratio. Then, aimed at the small area of bright light spots in the image, a light mask was used to reduce the influence of bright light on the extraction of effective information.

#### 2.2.2. Feature Extraction

As multispectral images involve multi-band spectral data, traditional vegetation indices are difficult to apply to feature extraction for all types of data. Therefore, deep-learning methods are introduced to extract data features based on pre-designed rules.

In view of the multispectral images collected in this paper, a vegetation-index method based on the fusion of improved band features and details was proposed. In order to better explore the differences in the reflectance of different features, multispectral images with rich information in the dataset were selected to count the image element values of cyanobacterial blooms, clean water bodies, floating vegetation, surface litter, boats and other materials in each waveband. Since the pixel values of cyanobacterial blooms and other interfering objects basically did not overlap in B1, B3 and B5, the multispectral feature-extraction method was verified based on the reflectivity of these three bands. NDVI is calculated based on the reflectance of B3 and B5 and is often used to detect vegetation growth and vegetation cover. The formula is shown in Equation (1):

(1) $$\mathrm{NDVI}=\frac{\rho_{B5}-\rho_{B3}}{\rho_{B5}+\rho_{B3}}$$

where $\rho_{B3}$ and $\rho_{B5}$ are the reflectance of the feature in the red and near-infrared bands, respectively. First, to keep the image features of the model input within the same range, the two-by-two combinations of wavebands were substituted into Equation (1) for normalization, with the goal of selecting the combination with the greater difference between the indices of each feature. In addition, to further extend the difference in indices between cyanobacterial blooms and other features, we adjusted the calculation method and enhanced the detail. Ultimately, this paper used the vegetation index which is given in Equation (2):

(2) $${\mathrm{NDVI}}_{\mathrm{sqrt}}=\mathrm{sgn}\left(\frac{\rho_{B5}-\rho_{B1}}{\rho_{B5}+\rho_{B1}}\right)\times \sqrt{\left|\frac{\rho_{B5}-\rho_{B1}}{\rho_{B5}+\rho_{B1}}\right|}$$

where $\rho_{B1}$ and $\rho_{B5}$ are the reflectance of the feature in the blue and the near-infrared wavelengths, respectively.

#### 2.2.3. Model Construction

As cyanobacterial blooms vary in size and shape and are mostly irregularly distributed, it is easy to miss some important image details during feature learning and misclassify pixel points at the junction of different objects or in some boundary areas. Therefore, on the basis of the improved vegetation index, this paper proposed an improved transformer model based on feature enhancement and region correction, achieving the following two improvements to robustly detect cyanobacterial blooms.

(1) Feature-Enhancement Module

In the data processing stage, the improved vegetation index was used to calculate the ${\mathrm{NDVI}}_{\mathrm{sqrt}}$ value of each pixel point within the image to form a single-channel image matrix, which was then converted into a three-channel two-dimensional matrix as the input to the network model. This paper also expanded the dataset to increase data diversity by randomly cropping and scaling the three-channel images whose tensor sizes were changed from 1600 × 1300 pixels to 768 × 768 pixels.

Boundary classification can mainly be achieved in two ways: one is to directly transfer the feature mapping of the shallow network in the model to the corresponding deep network, which is continuously transferred and superimposed; the other is to pool and convolve a certain network layer in the network structure for calculation. Both of the above methods are able to retain small amounts of spatial information, but the former greatly increases the computational load of the model, while the latter cannot guarantee the balance of the shallow-feature and deep-feature information to a certain extent. Aimed at these shortcomings, this paper designed a new feature-enhanced network architecture unit.

**Figure 2:**
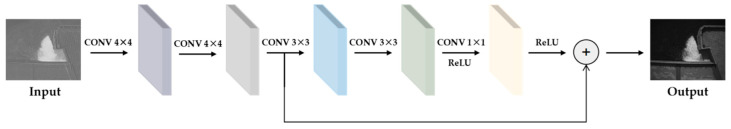
Structure of feature-enhancement module.

The design structure of the feature-enhancement module is shown in Figure 2. In order to extract more semantic features, to solve the problems of network degradation and gradient descent, and to improve the network performance, the feature-enhancement module designed in this paper contained five convolutional layers with one residual unit structure. Firstly, the stride of the first layer was chosen to be 4 and the padding to be 3. The image size was calculated to be 192 × 192 to further obtain the shallow features, and then used as the input for the next convolutional layer. Then, one convolution kernel with a size of 4 × 4 and two convolution kernels with a size of 3 × 3 were used to extract the multi-layer features of the image, respectively. In the residual structure unit, the 3 × 3 sized convolution kernel on the main channel was used to extract details of the image texture features, and the 1 × 1 sized one to obtain the deepest features of the graph. A layer of ReLU activation function was added between the two convolution layers to avoid gradient explosion and disappearance, allowing features in complex scenes to be learned more comprehensively. At the same time, the side channels transferred the edge features to the extracted feature maps, and the backbone network model contained rich spatial and semantic information by calculating pixel summation.

For a given multispectral feature image matrix, this feature-enhancement module performed several convolution calculations for different purposes to obtain rich, shallow and deep image features, respectively. The feature information was balanced to obtain multi-scale feature mapping, which not only helped to avoid feature loss caused by increased layers in the traditional deep-learning model, but also improved the convergence rate of training and obtained more comprehensive and rich multispectral image information.

(2) Region Correction Module

Pixel similarity is scored based on the similarity of information content between images, and the number of scores is used to determine the similarity of image information content, which is also used for image semantic segmentation. Ahn et al. [33] proposed a model framework to generate image-segmentation labels from a given image-level class label. In a weakly supervised environment, the training model can segment local discriminative parts instead of the whole object region. To correct the semantic information of adjacent pixels, this paper added a pixel-similarity-based region-correction module after the original model decoder, to identify the local region where the target object was located by estimating the semantic affinity between adjacent image coordinates. Then, pixel-level region correction was performed according to the predicted affinity to obtain accurate object location information.

Given a multispectral image, the corresponding Vector Class Activation Map (CAM) was calculated. The calculation method of the target class CAM is as in Equation (3) [33]:

(3) $$M_{c}\left(x,y\right)=W_{c}^{T}f^{cam}\left(x,y\right)$$

where $M_{c}$ is the vector class activation mapping, $W_{c}$ is the classification weight, and $f^{cam}\left(x,y\right)$ is the feature vector at the point on the feature map. The semantic similarity labels were generated based on the obtained CAM, which is calculated as in Equation (4):

(4) $$W\left(x_{1},x_{2}\right)=\{\begin{array}{c} 1,\;\;\;\;\;if\;l_{1}=l_{2}\\ 0,\;\;otherwise \end{array}$$

where $l_{1}$ and $l_{2}$ are the categories of the adjacent pixels $x_{1}$ and $x_{2}$, and $W\left(x_{1},x_{2}\right)$ is the pixel label. Within a certain radius, the neighborhood image was connected with adjacent pixels, and the semantic affinity of the connected pixel point pairs was calculated. The information about the pixel similarity of the edges in the image would be passed to semantically similar places by random traversal and to other similar places in the surrounding area by penalty. This semantic extension would significantly correct the CAM and recover the morphology of tiny objects, especially at the boundaries of different classes. Ultimately, the class tokens of the adjusted CAM were used to synthesize segmentation labels for training the segmentation model. The pixel-similarity-based region-correction module reduced the loss of pixel details, enhanced the information correlation and improved the accuracy of cyanobacterial-bloom detection in complex scenes.

#### 2.2.4. Evaluation Indicators

In order to evaluate the performance of the proposed algorithm and calculate the cyanobacterial bloom pollution area in the sampling area, the following evaluation indicators were used to for quantification.

Pixel Accuracy (*PA*) refers to the ratio of the number of pixels whose categories are correctly predicted by the algorithm to the total number of pixels, which can be understood as the percentage of correctly classified pixels. It is the most common and intuitive evaluation index in image segmentation [34,35], and its calculation is shown in Equation (5):

(5) $$PA=\frac{TP+TN}{TP+TN+FP+FN}$$

where *TP*, *FP*, *FN* and *TN* represent true positive, false positive, false negative and true negative cases, respectively. However, *PA* cannot determine whether the predicted cyanobacterial blooms are correctly located and may misclassify pixel points, not serving as a good indicator of the results in the case of unbalanced samples. Therefore, *PA* alone does not provide an accurate measure of algorithm performance. Intersection over Union (*IoU*) refers to the ratio of the intersection and union of the model’s prediction result and the true value for a certain category. It is a standard performance measure for object category segmentation problems [36] and is calculated using Equation (6):

(6) $$IoU=\frac{TP}{FP+TP+FN}$$

In the cyanobacterial-bloom detection task, *IoU* represents the magnitude of the difference between the algorithm’s predicted cyanobacterial blooms and the true value. The higher the value, the better the detection effect. This paper uses a combination of *PA* and *IoU* to assess the improvement in the improved vegetation-index method on the overall accuracy of the algorithm.

At the same time, the proportion of the area contaminated by cyanobacterial blooms can be estimated by counting the number of cyanobacterial-bloom pixels on the river surface, and the calculation formula is shown in Equation (7):

(7) $$P=\frac{A_{1}}{A}\times 100\%$$

where $A_{1}$ is the number of cyanobacterial-bloom pixels, and *A* is the total number of image elements.

## 3. Results

**Figure 3:**
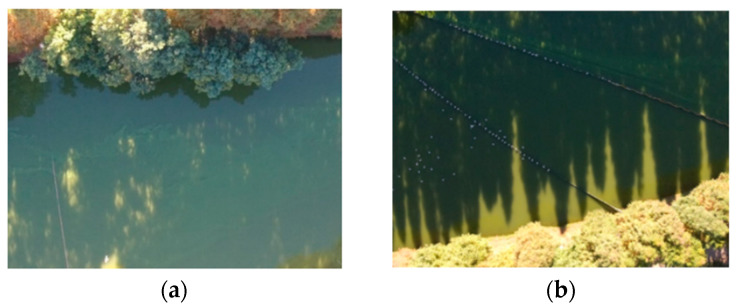
Complex river scenes: (**a**) reflection case; (**b**) shadow case.

In the detection of cyanobacterial blooms, the diversity of the river and lake environment—as evidenced by distracting factors such as birds, boats, reflections and shadows on the surface of the water body—can, to some extent, affect the detection accuracy of the algorithm; this makes the detection of cyanobacterial blooms very difficult. Figure 3 indicates the presence of irregularly sized light spots and large reflections on the river surface, respectively, which can change the pixel characteristics and, thus, lead to false or missed detections. In this paper, the randomly selected samples were divided into two categories—regular scenes and complex scenes—to test the performance of the algorithm. Among them, a regular scene refers to a situation wherein there is no obvious interference, and a complex scene indicates that there is interference dominated by strong light and reflections.

### 3.1. Results of Feature Extraction

For different scenes, we compared the feature extraction effects before and after the improved vegetation-index method, and input the different features extracted into the same transformer model to analyze and compare the model output results.

**Figure 4:**
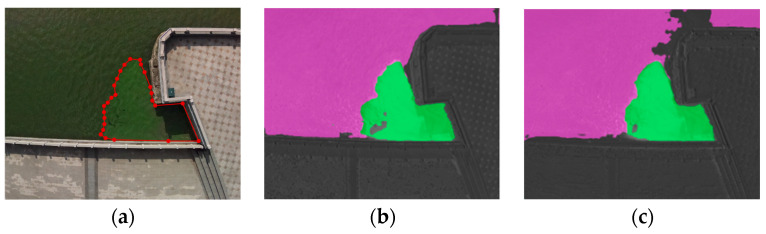
Detection results of different algorithms for regular scene: (**a**) visible light image; (**b**) detection results of NDVI as the feature-extraction method; (**c**) detection results of NDVI_sqrt_ as the feature-extraction method.

Figure 4a shows the original visible image in a typical regular scene of the Xujiang River in Suzhou. Figure 4b shows the detection result of the multispectral features extracted from NDVI as input to the transformer model, which can basically distinguish cyanobacterial blooms from clean water; however, there is some error in the detection effect of cyanobacterial blooms in the lower-concentration areas. Figure 4c shows the detection results of the multispectral features extracted based on NDVI_sqrt_ as the input of the transformer model. There is still a small amount of misclassification at the riverbank boundary, but the model has enhanced the learning of the spectral features of cyanobacterial blooms. It can be seen intuitively that compared with NDVI, NDVI_sqrt_ identifies cyanobacterial blooms at low concentrations or in the early stages of growth. Provably, NDVI_sqrt_ can improve detection accuracy by extracting cyanobacterial spectral features that are weaker and more difficult to identify.

**Figure 5:**
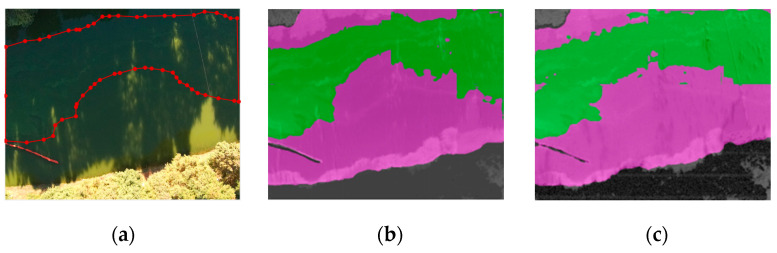
Detection results of different algorithms for reflection scene: (**a**) visible light image; (**b**) detection results of NDVI as the feature-extraction method; (**c**) detection results of NDVI_sqrt_ as the feature-extraction method.

Figure 5a shows the original visible image in a typical reflection scene of the Daqing River in Yunnan. The light spot in the lower right corner covers part of cyanobacterial bloom, and brings great difficulty to accurate detection. Figure 5b,c show the detection results using the multispectral features extracted before and after the improved vegetation index as the input of the transformer model, respectively. The former had obvious detection errors and missed a large area of cyanobacterial blooms; meanwhile, the latter was able to identify cyanobacterial blooms in the reflection scene, probably because NDVI_sqrt_ was able to extract more accurate spectral features, coupled with the stronger learning ability of the attention mechanism. High-precision detection of cyanobacterial blooms can still be achieved, even under strong light conditions.

**Figure 6:**
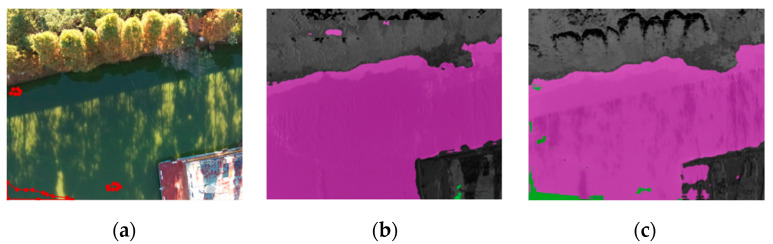
Detection results of different algorithms for shadow scene: (**a**) visible light image; (**b**) detection results of NDVI as the feature-extraction method; (**c**) detection results of NDVI_sqrt_ as the feature-extraction method.

Figure 6a shows the original visible image in a typical shadowy scene of the Daqing River in Yunnan. Figure 6b,c show the detection results of the multispectral features extracted before and after the improved vegetation index as the input of the transformer model, respectively. Both can distinguish the riverbank and water body more correctly. However, it can be seen that for mild cyanobacterial blooms under shadow coverage, NDVI extracted almost no features, and cyanobacterial blooms and partial vegetation were mistaken for water. On the contrary, NDVI_sqrt_ was able to practice efficient retrieval, even when covered.

A comparison of the detection performance of several feature-extraction methods for cyanobacterial blooms in different test scenarios is shown in Table 2. The detection indicators of several algorithms in conventional scenarios were not significantly different, the results were all around 80%. The accuracy of the cyanobacterial-bloom detection algorithm used in this paper was much higher than the above two methods in scenarios with reflections and shadows, reaching 86.4% and 87.6%, respectively, and the IoUs were as high as 0.79 and 0.82. Compared with the vegetation index as the model input before improvement, the accuracy improved by 11.1% and 5.4%, and the IoU was 0.15 and 0.03 higher, respectively. The effectiveness of NDVI_sqrt_ was proven to extract features, so it was applied in subsequent deep-learning models.

Among the more typical river channels tested, we studied the Daqing River and the Haigeng Park river channel; these are more complex river scenarios, with interference from boats, birds and litter on the river surface, in addition to strong light and reflections. A comparison of the detection performance of several feature-extraction methods for cyanobacterial blooms in different typical river channels is shown in Table 3. The algorithm proposed in this paper maintained a more stable detection effect in all four river channels, unaffected by external interference factors, and the average accuracy of cyanobacterial-bloom detection was above 85%, much higher than 70% before improvement. In Xujiang River, the detection accuracy increased from 85.3% to 92.3%, which extracted some information about cyanobacterial blooms in the early stage of growth. Moreover, in the Daqing River, Baoxiang River and Haigeng Park with different kinds of disturbances, the detection accuracy before and after using the improved vegetation index increased by 13.3%, 17.9% and 25.9%, respectively. The improvement in the intersection ratio showed that the overall classification task had a good effect, and the accuracy improvement caused by the misjudgment of the category was excluded. In general, the impressive improvement results support that NDVI_sqrt_ has great application value in practical scenarios.

### 3.2. Detection Results

To further verify the effectiveness of the improved transformer model, we used the improved vegetation index for feature extraction and compared the detection results of the improved transformer model with those of FCN and SegNet.

**Figure 7:**
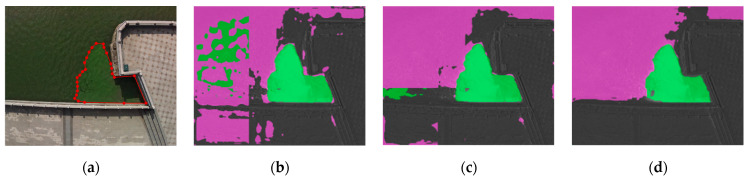
Detection results of different deep-learning algorithms for regular scene: (**a**) visible light images; (**b**) detection results of FCN; (**c**) detection results of SegNet; (**d**) detection results of improved transformer model.

Figure 7 shows a comparison of the detection results of the three deep-learning algorithms in conventional scenarios of the Xujiang River in Suzhou. There was a large number of misclassifications in the detection results of FCN, wherein the reflections on the water body, due to the fluctuations, were considered to be cyanobacteria, and some riverbanks were also mistaken for water bodies. The overall detection effect of FCN was very bad. Although there were relatively fewer false detections in SegNet than in FCN, the classification was not clear, with the boundaries of water bodies, cyanobacteria, and riverbanks all misclassified among each other. In comparison, the algorithm based on the improved transformer model was more accurate for cyanobacterial blooms and had almost no false detections.

**Figure 8:**
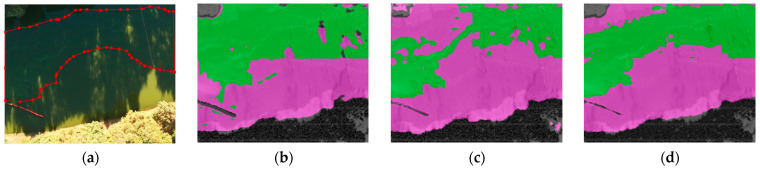
Detection results of different deep-learning algorithms for reflection scene: (**a**) visible light images; (**b**) detection results of FCN; (**c**) detection results of SegNet; (**d**) detection results of improved transformer model.

Figure 8 shows a comparison of the detection results of the three deep-learning algorithms in the reflection scene of the Daqing River in Yunnan. Both FCN and SegNet had false and missed detections. FCN was more accurate in detecting the category of riverbanks, but some water bodies were judged to be cyanobacterial blooms, thus estimating a larger pollution area. Meanwhile, SegNet did not accurately distinguish riverbanks from clean water bodies, and some cyanobacterial blooms were identified as water bodies, causing the pollution area estimate to be smaller. The interference of reflections was severe, which caused the pixel value of the corresponding position to change so that the above two algorithms did not work correctly. In contrast, the algorithm proposed in this paper could effectively achieve accurate detection of cyanobacterial blooms in these edge areas, even under strong light interference.

**Figure 9:**
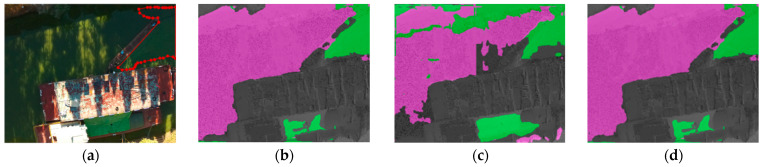
Detection results of different deep-learning algorithms for shadow scene: (**a**) visible light images; (**b**) detection results of FCN; (**c**) detection results of SegNet; (**d**) detection results of improved transformer model.

Figure 9 shows a comparison of the detection results of the three deep-learning algorithms in the shadowy scene of the Daqing River in Yunnan. The accuracy of FCN was slightly affected, and SegNet misidentified a large number of water bodies as cyanobacterial blooms. Such detection results were contrary to misjudgments caused by reflections; this could infer that FCN can exclude shadows interference, while SegNet can exclude reflection interference. Contrarily, the improved transformer model maintained high-accuracy detection in all scenarios. For objects of a certain color on the ship, all three algorithms misjudged, which was not an error caused by the boundary problem.

Both PA and IoU were still used here to quantitatively evaluate the detection performance of several image-segmentation algorithms. The comparison of the detection performance of cyanobacterial blooms in different test scenarios is shown in Table 4. The difference in detection performance in conventional scenarios was not significant, but the difference in complex scenarios was obvious. FCN was the least satisfactory, with a recognition accuracy of 61% and 59.3% under reflections and shadows, respectively. SegNet had a certain degree of false detection and missed detection at the boundaries of different features, and the detection accuracy was basically maintained at 60–65%. Compared with FCN and SegNet, the average detection accuracy of the improved transformer model in this paper reached over 85% and the IoU was around 0.8, while the detection accuracy levels of FCN and SegNet were only 64.1% and 67.3%.

### 3.3. Estimation of Contaminated Area and Determination of Contamination Level

According to the quantitative assessment method of algal blooms in the Technical Specification for Remote Sensing and Ground Monitoring and Evaluation of Algal Blooms promulgated by the Ministry of Ecology and Environment of China in 2020 [37], this paper used the proportion to evaluate the degree of cyanobacterial-bloom pollution, which was classified into five levels from light to heavy: I, II, III, IV and V, as shown in Table 5.

**Figure 10:**
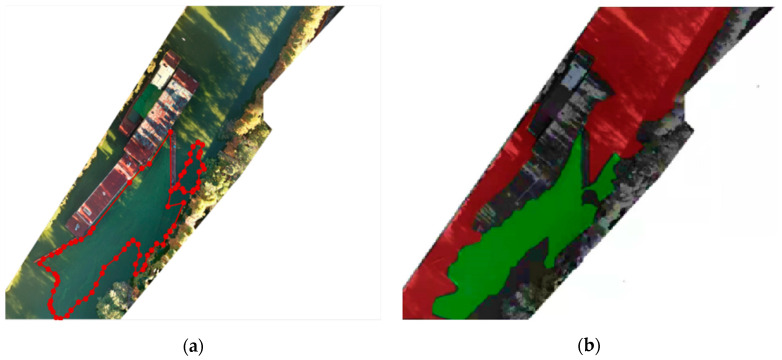
Daqing River Example of engineering application: (**a**) visible light image, the area surrounded by red dots is cyanobacterial blooms; (**b**) detection result of our algorithm.

In this paper, we used DJI SmartMap software to stitch together the Daqing River, Xujiang River, Jinji Lake and Baoxiang River to estimate the proportion, and judged the pollution level of cyanobacterial blooms. Taking the Daqing River as an example for demonstration, Figure 10a shows the visible image of the Daqing River after stitching, and Figure 10b shows the detection results of the algorithm in this paper.

As can be seen from Table 6, in the rivers with neatly shaped river surfaces, a single detection category and clearly distinguishable feature categories, such as the Xujiang River, both NDVI and the algorithm in this paper had good detection results whose detection errors were within acceptable limits. However, in scenes with complex scenarios wherein interference was serious and regional boundaries were unknown, the detection accuracy of the algorithm in this paper was much higher than that of NDVI. This was especially the case for the Daqing River, for which NDVI had a misjudgment rate of 18%, which directly led to the wrong judgment of pollution level from IV to V.

## 4. Discussion

### 4.1. Low-Altitude Remote-Sensing Technology

The conventional method of field sampling combined with laboratory analysis has insufficient capabilities for detecting cyanobacterial blooms in large-scale river channels. Owing to the influence of cloud cover, effective data for lakes and rivers from remote-sensing satellites are limited, and it is impossible to accurately monitor cyanobacterial blooms in small rivers in real time. However, low-altitude imaging remote-sensing technology can flexibly acquire multi-scene data according to demand. This technology does not require atmospheric correction of image data, but it will certainly magnify spatial interference such as shadows, lighting, and floating objects from sky light and the water’s surface, so certain preprocessing work is required. Low-altitude remote-sensing technology also makes it possible to monitor daily changes in cyanobacterial blooms, which can provide more real-time information for early warnings and salvage treatments of cyanobacterial blooms.

### 4.2. Improved Vegetation Index

The spectral characteristics of water bodies are determined by the absorption and scattering properties of optical radiation. The chlorophyll content in water increases rapidly, resulting in changes in its spectral characteristics when cyanobacterial blooms occur. The absorption peaks and reflection peaks of different wavelength bands of reflection spectral characteristics are different, so the vegetation-index method aims to judge the cyanobacterial blooms and distinguish other objects by calculating the reflectivity matrix of specific bands. Some research groups have reported improved vegetation indices and applied them to different river scenarios [16,17,18,19]. Fernandez et al. [38] compared 26 vegetation indices and concluded that NDVI is effective and the most widely used method. However, it is difficult to have a universal index, because there are many factors that can cause changes in reflectivity, such as: sun position, sensor position, geographic location, seasons, climate changes, humidity changes, variability of the ground objects themselves, and atmospheric conditions. The selection of vegetation index is affected by the type of experimental data. As shown in the results section, NDVI_sqrt_ has an accuracy of more than 85%, which is 5% higher than the 79.60% of the reference [39]. Compared to other indices, the improved vegetation index is more suitable for the feature extraction of cyanobacterial blooms in this paper.

### 4.3. Improved Transformer Model

Deep learning is a powerfully adaptive method with many achievements in image classification. It is necessary to introduce semantic segmentation for cyanobacterial-bloom detection, because cyanobacterial blooms are mostly granular, appearing as flocculent suspended matter, and the distribution shape is irregular. The detection accuracy of reference [29] is 93.68%, but the scene is simple and clear. Reference [30] achieves an average accuracy of 86.70% under complex lighting conditions. The improved transformer model has a test accuracy of 89.4% in the Daqing river with serious interference, which shows the effectiveness of the proposed algorithm in this paper.

As shown in Figure 4, this paper also tests the common semantic segmentation models CNN and DNN. FCN is insensitive to details and the size of the pixel blocks is much smaller than the whole image, which is not fully applicable to unstructured data. SegNet ignores adjacent information, and image resolution is reduced during network processing; thus, features are not sufficiently trained, causing interference to the contextual semantic understanding. To enhance the understanding of contextual semantics and exploit the multispectral features of images more effectively, the transformer model based on feature enhancement and region correction in this paper pays attention to the associations between adjacent pixels, and uses a multi-headed attention mechanism, which improves the classification accuracy of cyanobacterial-bloom boundary regions.

## 5. Conclusions

This work introduced a multispectral detection model for cyanobacterial blooms based on deep learning. Images were preprocessed with adaptive median filtering and light masks. The traditional vegetation-index method was improved to extract the multispectral features of cyanobacterial blooms, and then the transformer neural network was used for the first time to perform pixel-level semantic recognition of cyanobacterial blooms. Finally, the algorithm was applied to multiple rivers to accurately evaluate their pollution area and pollution levels. This method not only greatly reduced the sampling time, but also improved the accuracy and efficiency of the cyanobacterial-bloom detection algorithm, providing the possibility for online monitoring of cyanobacterial blooms. The research results of this paper contribute to the fields of water-quality anomaly detection, remote-sensing technology and deep learning. On the one hand, the improved vegetation index showed a better feature extraction effect than other indices in the dataset of this paper, and may expand the applicability of vegetation indices in the field of low-level multispectral cyanobacterial-bloom monitoring. On the other hand, the transformer neural network was first applied in the detection of cyanobacterial blooms, and showed good results when modifying the corresponding network structure. The work of this paper has certain enlightenment significance for the application of the transformer model in the multispectral detection of cyanobacterial blooms, and provids strong support for the application of neural networks in the field of environmental monitoring.

For future work, research on preprocessing methods can be introduced to eliminate insignificant environmental interference before feature extraction. In addition, higher sampling frequency may reveal the trends of cyanobacterial blooms, and a corresponding early-warning model, with the help of time series analysis, is recommended to develop a method of predicting future growth trends of cyanobacterial blooms; this is significant for early warnings and the rapid treatment of cyanobacterial blooms.

## Figures and Tables

**Table 1:** Distribution of cyanobacterial blooms datasets.

Province and City of Sampling	Specific Locations	Number of Images/Sheet
Kunming, Yunnan	Daqing River	708
	Baoxiang River	1080
	Haigeng Park	558
Suzhou, Jiangsu	Xujiang River	1782
	Jinji Lake	564
	Youlian New Village	108
Huzhou, Zhejiang	Taihu Lake	510
	Maoer Port	90

**Table 2:** Comparison of the detection performance of different feature-extraction methods for cyanobacterial blooms under various scenarios.

Scenes	NDVI		NDVI_sqrt_	
	PA	IoU	PA	IoU
Regular	79.5%	0.71	81.6%	0.76
Reflections	75.3%	0.64	86.4%	0.79
Shadows	82.2%	0.79	87.6%	0.82

**Table 3:** Comparison of the performance of different feature-extraction methods for the detection of cyanobacterial blooms in typical river channels.

River	NDVI		NDVI_sqrt_	
	PA	IoU	PA	IoU
Daqing River	76.1%	0.64	89.4%	0.76
Baoxiang River	63.4%	0.58	81.3%	0.86
Xujiang River	85.3%	0.85	92.3%	0.91
Haigeng Park	56.4%	0.57	82.3%	0.76

**Table 4:** Comparison of the detection performance of different deep-learning methods for cyanobacterial blooms in various scenarios.

Scenes	FCN		SegNet		Improved Transformer	
	PA	IoU	PA	IoU	PA	IoU
Regular	72%	0.75	75.4%	0.72	87.6%	0.82
Reflections	61%	0.56	60.9%	0.62	81.6%	0.76
Shadows	59.3%	0.62	65.7%	0.73	86.4%	0.79

**Table 5:** Grading standard of degree of cyanobacterial bloom based on the evaluation of pollution area ratio.

Levels	Proportion—P (%)	Characteristics
I	0	No cyanobacterial blooms
II	0 < P < 10	No obvious cyanobacterial blooms
III	10 ≤ P < 30	Mild cyanobacterial blooms
IV	30 ≤ P < 60	Moderate cyanobacterial blooms
V	60 ≤ P ≤ 100	Severe cyanobacterial blooms

**Table 6:** Estimated area of cyanobacterial-bloom contamination and determination of pollution level in typical rivers.

River	NDVI		Proposed Method		True Value	
	Percentage	Level	Percentage	Level	Percentage	Level
Daqing River	65.9%	V	48.7%	IV	47.9%	IV
Xujiang River	20.2%	III	21.8%	III	20.5%	III
Jinji Lake	91.6%	V	77.6%	V	79.4%	V
Baoxiang River	85.2%	V	79.6%	V	78.9%	V

## Data Availability

Not applicable.
